# Stemness and Survival: CD117^+^/CD133^+^ Subpopulations Sustain PI3K Signaling and Drive Imatinib Resistance in Head and Neck Mucosal Melanoma

**DOI:** 10.3390/cells15080721

**Published:** 2026-04-19

**Authors:** Sofie-Yasmin Hassan, Simeon Santourlidis, Thomas W. Flanagan, Sarah-Lilly Hassan, He Zhou, Morna F. Schmidt, Claudio Cacchi, Matthias Ferdinand Lammert, Mossad Megahed, Amir Sadegh Yazdi, Danny David Jonigk, Marcos J. Araúzo-Bravo, Robert T. Brodell, Sybille Facca, Youssef Haikel, Mohamed Hassan

**Affiliations:** 1 Department of Pharmacy, Faculty of Mathematics and Natural Science, Heinrich-Heine University of Düsseldorf, University Street 1, 40225 Duesseldorf, Germany; 2 Institute for Transplantation Diagnostics and Cell Therapeutics, University Hospital of Duesseldorf, 40225 Duesseldorf, Germany; 3 Department of Pharmacology and Experimental Therapeutics, LSU Health Sciences Center, New Orleans, LA 70112, USA; 4 Department of Pharmacy, University of Bonn, An der Immenburg 4, 53121 Bonn, Germany; 5 Department of Internal Medicine, School Medicine, Tulane University, New Orleans, LA 70112, USA; 6 Department of Dermatology and Allergology, University Hospital RWTH Aachen, 52074 Aachen, Germany; 7 Institute of Pathology, University Hospital of Aachen, 52074 Aachen, Germany; ccacchi@ukaachen.de (C.C.);; 8 German Center for Lung Research, BREATH Hanover, 30625 Hanover, Germany; 9 Center for Integrated Oncology Aachen Bonn Cologne Düsseldorf (CIO ABCD), 52074 Aachen, Germany; 10 Group of Computational Biology and Systems Biomedicine, Biogipuzkoa Health Research Institute, 20014 San Sebastián, Spain; mararabra@yahoo.co.uk; 11 Ikerbasque, Basque Foundation for Science, 48013 Bilbao, Spain; 12 Department of Cell Biology and Histology, Faculty of Medicine and Nursing, University of Basque Country (UPV/EHU), 48940 Leioa, Spain; 13 Department of Pathology and Dermatology, University of Mississippi Medical Center, 2500 North Sae Street, Jackson, MS 39216, USA; 14 ICube CNRS UMR7357, University of Strasbourg, 67000 Strasbourg, France; 15 Institut National de la Santé et de la Recherche Médicale, University of Strasbourg, 67000 Strasbourg, France; youssef.haikel@unistra.fr; 16 Department of Operative Dentistry and Endodontics, Dental Faculty, University of Strasbourg, 67000 Strasbourg, France; 17 Pôle de Médecine et Chirurgie Bucco-Dentaire, Hôpital Civil, Hôpitaux Universitaire de Strasbourg, 67000 Strasbourg, France; 18 Research Laboratory of Surgery-Oncology, Department of Surgery, Tulane University School of Medicine, New Orleans, LA 70112, USA

**Keywords:** HNMM, CSCs, c-Kit, p85, PI3K, imatinib

## Abstract

**Highlights:**

**What are the main findings?**

- Like cutaneous melanoma, head and neck mucosal melanoma contains a subpopulation of cancer stem-like cells.
- Head and neck mucosal melanoma is characterized by the expression of stemness markers including CD20, CD117, CD133 and CD166 proteins.

**What are the implications of the main findings?**

- In addition, the stemness properties CD117^+^/CD133^+^ of head and neck mucosal melanoma subpopulations confer resistance to the specific inhibitor of c-Kit mutation.
- In head and neck mucosal melanoma, the CD117 signal to PI3K/PDK1/AKT/NF-κB and PI3K/PDK1/AKT/MDM2 pathways is essential to preserve the stem cell properties and neutralize drug toxicity.

**Abstract:**

Head and neck mucosal melanoma (HNMM) arises in the nasal and oral cavities and has the propensity to metastasize to local and distant body sites. HNMM is also notable for its resistance to available therapeutics. The rarity of this disease makes it difficult to conduct large-scale clinical studies to develop standard treatment protocols. In contrast to cutaneous melanoma, c-Kit-dependent pathways are well studied in HNNMM and provide a potential therapeutic target. We identified and isolated genetically distinct subpopulations with stem cell characteristics in HNMM samples bearing Kit wild-type and mutations. Functional analysis of these subpopulations reveals that, in addition to expressing the stem cell marker proteins CD20, CD117, CD133, and CD166, these subpopulations are characterized by self-renewal potential, migratory capacity, and resistance to Kit inhibitors such as Imatinib. Immunofluorescence staining and inhibition experiments demonstrate that the maintenance and resistance of HHMM subpopulations to Kit inhibitors is mediated by the Kit signal to the PI3K signaling pathway. The KIT signal to the PI3K signaling pathway does not result exclusively from a KIT mutation localized to Exon 17, but can also be triggered by mutations localized to Exons 11 and 13. In the present study, we identify and characterize an HNMM subpopulation with stemness properties in patients with c-Kit wild-type and mutation, and demonstrate for the first time the mechanisms by which the CD117^+^/CD133^+^ HNMM subpopulations survive and confer resistance to the specific inhibitor of c-Kit mutation.

## 1. Introduction

Head and neck mucosal melanoma (HNMM) is an extremely rare malignancy representing 0.2–8% of all melanomas in the United States and Europe [1]. It originates from melanocytes located in the mucosa of the oral and nasal cavities [2,3]. While the overall incidence of cutaneous melanoma (CM) is lower in darkly pigmented populations, the relative frequency of HNMM is higher in this group than in caucasians [3]. Patients with HNMM exhibit the lowest five-year survival among all melanoma subtypes [4]. The 5-year overall survival rate for mucosal melanomas of the head and neck is low (20% to 38%) [5,6]. Studies report rates below 30%, which are attributed to including patients with an aggressive disease in the advanced stage [7,8,9]. Any delay in starting treatment also plays a significant role due to the tendency for early hematogenous metastasis in HNMM [10]. Surgery and radiation therapy are the main treatment options, but once HHMM has metastasized, treatment becomes extremely difficult [11]. The subpopulations of HNMM demonstrate variations in phenotype and biology [12,13]. In fact, there is heterogeneity even within the same tumor subtype in a single patient (intratumoral heterogeneity) [13,14].

Although all melanocytes share the same embryonic origin, their function and fate are controlled by the microenvironment [15]. Consequently, the pathophysiology of the mucosal melanoma (MM) differs from those of other melanoma subtypes. While the RAS-RAF-MEK-ERK pathway is functionally significant for the development, progression, and resistance of CM [16], autocrine stimulation of the PI3K signaling pathway by c-Kit has also been studied in multiple myeloma where it is considered to play a role in tumor growth and treatment resistance. Furthermore, this pathway represents a potential therapeutic target [17,18,19]. Similar to its role in the progression of CM and treatment resistance, PI3K is expected to play a significant role in the pathophysiology of HNMM [20].

CD117 (c-Kit) is a transmembrane tyrosine kinase receptor that is commonly expressed in mucosal melanomas and is reported to be expressed in up to 78–81% of analyzed cases [21,22]. Although CD117 expression in mucosal melanoma is high, not all CD117-positive tumors have a corresponding KIT mutation [23,24]. KIT mutations have been described in 15–20% of mucosal melanoma cases and their significant role in mucosal melanomas is well documented [18,25].

As has been widely documented, CD20 expression is low in both CM and HNMM [26,27], and CD20^+^ cells represent a small subset of about 2% of melanoma cells with stem-cell-like properties [28]. Also, the contribution of CD20^+^ cells to tumorigenesis, resistance and metastasis has been reported [29]. Thus, targeted treatment of these CD20^+^ cells with anti-CD20 antibodies such as rituximab has shown promising results in preclinical and pilot studies and could reduce the risk of recurrence and improve overall survival in advanced and chemotherapy-resistant melanoma [29,30].

Prominin-1/AC133 (CD133), encoded by the PROM1 gene, belongs to the family of pentaspan transmembrane glycoproteins [31,32,33]. CD133 is specifically localized to cellular membranes with an extracellular N-terminal domain, 5-transmembrane domains separating two large glycosylated extracellular loops, two small intracellular loops, and an intracellular C-terminal domain [31,33]. In addition to serving as a stem cell marker, the CD133 protein is involved in the regulation of stem cell maintenance and drug resistance via PI3K-dependent mechanisms [19,34].

CD166, the activated leukocyte cell adhesion molecule (ALCAM), is a transmembrane protein that acts as a progression marker in melanoma and is also associated with increased invasiveness, metastasis, and poor prognosis [35]. Increased expression of CD166 has been reported to correlate with deep dermal invasion and, particularly in oral mucosal melanoma, with extensive vascular invasion [36,37].

Like those of many tumor types, cancer stem cells (CSCs) of HNMM are characterized by their unique protein signature and aberrant signaling routes [38]. We identify and characterize HNMM stem cells (HNMMSCs) in patients’ specimens and demonstrate that the c-Kit signal to PI3K and its downstream signaling pathways is responsible for the maintenance and drug resistance of HNMMSCs.

## 2. Materials and Methods

### 2.1. Immunogenetic Separation (IMS)

CD117^+^/CD133^+^ subsets of CSCs were isolated from surgery-resected human HNMM tissues. CD117^+^/CD133^+^ subpopulations were prepared from consenting patients with fresh HNMM, after institutional review board protocol approval (IRB) of the University Hospital of Aachen, Germany. HNMM samples were obtained from patients (*n* = 3). The first tumor lesion (P1) derived from an oral mucosal melanoma diagnosed as a primary oral mucosal melanoma of the hard palate (asymmetry) and anterior palate (Board), and exhibits BRAF, NRAS, and Kit wild-type. The second tumor lesion (P2) is oral mucosal melanoma that is diagnosed as hard palate (Asymmetry), anterior palate (Board) and has variations in color and harbors wild-type BRAF, wild-type NRAS and Kit-activating mutation (D820E). The third lesion (P3) is derived from nasal mucosal melanoma that is diagnosed as nasal septum evolving (E) change in color and shape/nasal sinus larger than 6 mm in diameter (D) harboring wild-type BRAF, NRAS-activating mutation (Q61K) and wild-type cKIT. The preparation of specimens of P1, P2 and P3 was performed within 1 to 2 h after surgical removal; tumors were rinsed and trimmed to remove connective tissues before mechanical–enzymatic dissociation to prepare a single-cell suspension using mechanical mincing and enzymatic digestion using the human Tumor Dissociation Kit MACS (#130-095-929) Miltenyi Biotech, Mönchengladbach, Germany, as described [19,39], followed by Magnetic Depletion after the incubation with the dissociated cell suspension with antibody-conjugated microbeads targeting contaminating cells: anti-CD45 (leukocytes), anti-CD31 (endothelial cells), and optional anti-CD90 (fibroblasts) using the human Lineage Cell Depletion Kit (#130-092-211), all from Miltenyi Biotech, Mönchengladbach, Germany. Then, the unlabeled flow-through containing the enriched HHNMM cells were then washed twice with PBS containing 5% FCS before cultivation for two weeks in collagen-coated Petri dishes using low-serum melanocyte growth media (Millipore Sigma) supplemented with growth factors like Phorbol 12-myristate 13-acetate (PMA), bovine pituitary extract (BPE), insulin, and fibroblast growth factor (FGF). Then the cells were subjected to the purification of CD117^+^/CD133^+^ and CD117^−^/CD133^−^ subpopulation by MACS (Miltenyi Biotech, Mönchengladbach, Germany), as described [39]. This study was first approved for the time from 2015 to 2024 by the Institutional Review Board (IRB) of [University hospital of Aachen] (Protocol No.: 25-137), and the extension of the former Approval was confirmed on: 28 March 2025. All participants provided written informed consent prior to inclusion, and the study was conducted in accordance with the Declaration of Helsinki.

### 2.2. Sphere Formation Assay

The sphere formation assay was performed as described [19,20,31].

### 2.3. Wound Healing Assay

Wound-healing assay was performed as described [19].

### 2.4. Cell Viability Assay

Determination of cell viability was performed with the MTT assay as previously described [40].

### 2.5. Reagents, Inhibitors, siRNAs and Antibodies

Imatinib mesylate was provided by Novartis (Basel, Switzerland) and was used at a concentration of 10 µM. Dasatinib, from Bristool Myers Squuib, Princeton, NJ, USA, was used at a concentration of 1 µM. The potent inhibitor of PI3K, LY294002 (MedKoo Cat#: 201795) from Medkoo Biosciences Inc. (Durham, NC, USA) and was used at a concentration of 10 µM. The potent inhibitor of AKT, AKT inhibitor VIII (Cat.: A3149), from APExBio and was used at a concentration of 200 nM. The potent inhibitor of PDK1, BX517 (Cat.: B5838), from APExBio (Houston, TX, USA) and was used at a concentration of 1 µM. The PI3K p85α shRNA (SC-36217-V) and c-Kit siRNA (sc-29225) are all from Santa Cruz (Dallas, TX, USA). All reagents were obtained from Sigma-Aldrich (St. Louis, MO, USA) unless otherwise indicated. The CellTiter 96 AQueous One Solution Cell Proliferation Assay (MTS) was purchased from Promega (Madison, WI, USA). All antibodies for Western blot, immune fluorescence (IF) and immune histochemistry, including anti-CD117/c-Kit [(D13A2) XP^®^ Rabbit mAb #3074], CD133 [(D2V8Q) XP^®^ Rabbit mAb #64326], anti-CD133 (#64326), c-Kit (#37805), p85 (#4257), and Actin (#8457), and Phospho-PDK1 [Ser241] (#3061) were all purchased from Cell Signaling Technology (Beverly, MA, USA); Anti-PDK1 antibody [EPR19571] (ab202468) Abcam, Cambridge, UK. Phospho-AKT (Ser473) antiobody (Cat. Nr.: 80455-1-RR) and anti-AKT antibody (Cat. Nr.: 10176-2-AP) were purchased from Proteintech Group, Inc., Rosemont, IL, USA.

### 2.6. Western Blot

Immunoblot analysis was performed according to standard procedures [19]. The following antibodies were used at the indicated dilution: Anti-CD117/c-Kit (#3074]), 1:1000; anti-CD133 (#64326), 1:1000, and anti-p85 (SC-71891), 1:1000; anti-PARP (#9542), 1:500; all from Cell Signaling Technology Inc., Danvers, MA, USA; anti-Actin 1:5.000 antibody, anti-p53 (SC-47698), 1:1000; MDM2 (SC-965), 1:1000, all from Santa Cruz Biotechnology Inc., Santa Cruz, CA, USA. Anti-PDK1 antibody (ab202468), 1:500; anti-CD166 (ab109215), 1:1000; anti- Tubulin (ab68193), 1:1000 antibodies all from Abcam, Cambridge. Phospho-AKT (Ser473) (Cat. Nr.: 80455-1-RR) 1:500; anti-AKT (Cat. Nr.: 10176-2-AP), 1:1000 antibodies all from Proteintech Group, Inc., Rosemont, IL, USA.

### 2.7. Immunofluorescence Staining

Both CD117^+^/CD133^+^ and CD117^−^/CD133^−^ HNMMM supopulations were allowed to grow for 24 h, then cells were subjected to immunofluorescence staining as described in [19]. Primary antibodies; anti-CD117 (SC-2697), 1:200; and anti-p85 (SC-11415), 1:200 (Santa Cruz Biotechnology Inc., Santa Cruz, CA, USA) were allowed to bind overnight at 4 °C. Subsequently, cells were washed three times in PBS and incubated with Alexa Flour-labeled secondary antibodies. After three additional washes in PBS, cells were mounted using the DAKO mounting medium. Photomicrographs were taken on a fluorescence microscope (Leica, Wetzlar, Germany).

### 2.8. Immunohistochemistry

All specimens were embedded in an optimal cutting temperature compound (TissueTek) and frozen. Block sections (4 μm) were obtained with a criotome (Leica 1720 rotary criotome, Nussloch, Germany) and placed on slides. The detection of CD20, CD117, CD133 and CD166 was performed using the anti-CD20 antibody (1:200; CD20 antibody #GTX29475, Gentex) and the detection of melanoma was performed using the anti-Melanoma antibody Melan-A Antibody (A103): sc-20032)), 1:200 and CD117 antibody # 84259-5 proteintech, (1:200) anti-CD133 and CD166 antibodies (ab235957), and abcam (1:200), using the biotin/streptavidin/peroxidase methods.

### 2.9. Electrophoretic Mobility Schift Assay (EMSA)

The details of the electrophoretic mobility shift assay (EMSA) have been described [41]. The double-stranded synthetic oligonucleotides carrying a binding site for NF-κB (Santa Cruz Biotechnology Inc., Santa Cruz, CA, USA) were end-labeled with [γ-^32^P] ATP (Hartmann Analytika, Munich, Germany) in the presence of T4 polynucleotide kinase (Genecraft, Münster, Germany). The competition assay was performed in the same manner, except that unlabeled probes containing a NF-κB sequence were incubated with nuclear extracts for 20 min before adding the labelled probes. Visualization of bands was performed following electrophoresis and exposure to high-performance autoradiography film.

### 2.10. Mice

Athymic nu/nu nude mice (Charles River Lab., Inc., USA), 4–6 weeks old, were used in this study. Animals were housed in a barrier facility on a high efficacy particulate arrestee (HEPA)-filtered rack under standard conditions of 12 h light/dark cycles. The animals were fed an autoclaved laboratory rodent diet. All mouse surgical procedures and imaging were performed with the animals anesthetized by subcutaneous injection of a ketamine mixture (0.02 mL solution of 20 mg/kg ketamine, 15.2 mg/kg xylazine, and 0.48 mg/kg acepromazine maleate). The response of animals during surgery was monitored to ensure adequate depth of anesthesia. The animals were observed daily and humanely sacrificed by CO_2_ inhalation if they met the following endpoint criteria: severe tumor burden (more than 20 mm in diameter), prostration, significant body weight loss, difficulty breathing, rotational motion and body temperature drop. All animal procedures were approved by the Tulane University school of Medicine’s Institutional Animal Care and Use Committee (IACUC) and conducted under the authority of the Project License (A4499-01) according to the NIH Guide for the Care and Use of Laboratory Animals.

### 2.11. Imatinib Treatment

Mice were treated with either PBS or Imatinib (provided by Novartis, Basel, Switzerland) prepared in distilled water and dosed by intraperitoneal injection at 50 mg/kg/mouse, twice a day, for 42 consecutive days. Tumor size was measured at regular time intervals up to 4 weeks.

### 2.12. Statistical Analysis

All statistical analyses were performed using the GraphPad Prism 5 software (GraphPad, La Jolla, CA, USA). Data are presented as mean ± standard deviation (SD) of three independent experiments, each performed in triplicates. *P* < 0.05 was considered statistically significant.

## 3. Results

### 3.1. Identification of Head and Neck Mucosal Melanoma Subpopulations with Stemness Properties

**Figure 1:**
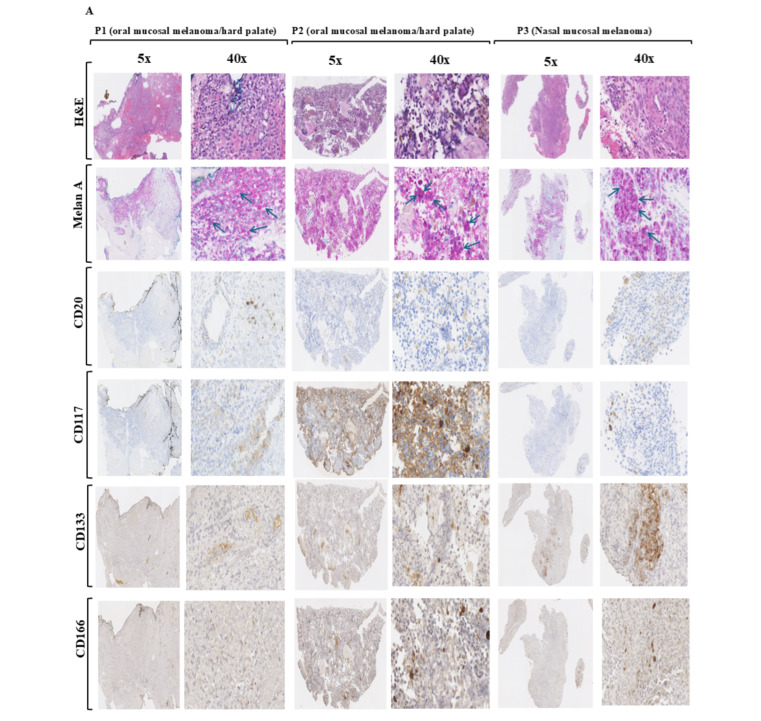

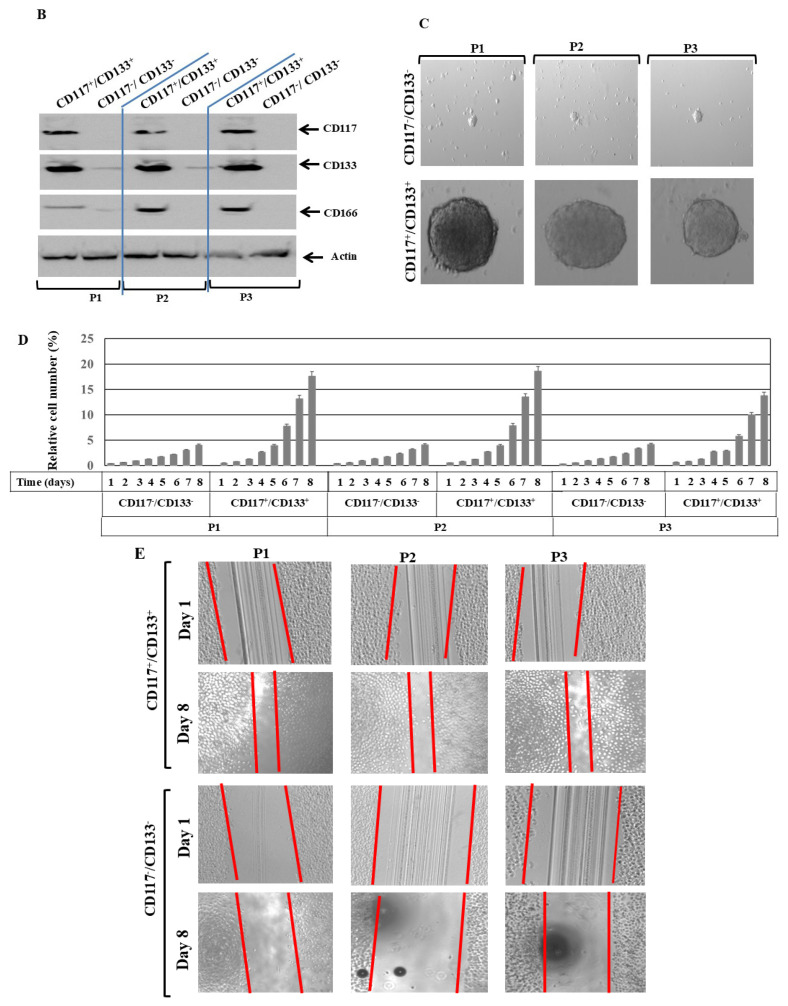
**Identification ad functional analysis of HNMM subpopulations.** (**A**) **Immune histochemistry**. HNMM tissues derived from oral and nasal mucosal melanoma lessions were stained with H&E to provide detailed cellular and tissue architecture visualization, and with anti- Melan A, a specific marker for melanoma, with anti-CD20, anti-CD117, anti-CD133 and anti-CD166 antibodies, a specific marker for CSCs (**B**) **Western blot analysis:** Detection of stem cell markers (CD117, CD133) by Western blot in sorted CD117^+^/CD133^+^ and CD117^−^/CD133^−^ cells derived from HNMM specimens (P1, P2 and P3) using specific antiobodies. Actin was used as internal control for loading and transfer. Data are representative of three independent experiments performed separately. (**C**) **Sphere-formation assay**: Single-cell formation assay demonstrates the abilty of freshly isolated CD117^+^/CD133^+^ and CD117^−^/CD133^−^ cells derived from HNMM specimens to generate in vitro tumor-like spheres. All images were captured on day 14. Data are representative of three indpendent experiments performed seprately. (**D**) **Cell viability assay**: Data of cell viability assay demonstrates the growth rate of CD117^+^/CD133^+^ and CD117^−^/CD133^−^ cells derived from HNMM specimens. Data means ± SD of three independent experiments performed in triplicate. (**E**) **Wound-healing assay**: Data of wound-healing assay demonstrate the migration potential of CD117^+^/CD133^+^ and CD117^−^/CD133^−^ cells derived from HNMM specimens. Data are representative of three independent experiments. The images were captured on day 1 and day 8.

First, we focused on the detection of CD133-positive cells in HNMM lesions (*n* = 3) to assess the portion of CSCs in the whole tumor by the analysis of CD20, CD117, CD133 and CD166 proteins in HNMM lesions as shown (Figure 1A). Then, we isolated and purified CD117^+^ and CD133^−^ HNMM subpopulations from HNMM lesions of pateint 1 (P1), patient 2 (P2) and patient 3 (P3) using our established protocol [19,39]. The expression of the stem cell marker proteins CD117, CD133, and CD166 was confirmed in the CD117^+^/CD133^+^ HHMM, but not in CD117^−^/CD133^−^ subpopulations using Western blot analysis (Figure 1B). Furthermore, CD117^+^/CD133^+^ subpopulations possess the potential to grow in a spheroid form, compared to CD117^−^/CD133^−^ subpopulations cultured in a serum-free, growth-factor-containing, anchorage-independent medium. Evidence of the self-renewal capacity of the CD117^+^/CD133^+^ HNMM subpopulations was observed within two weeks (Figure 1C). The self-renewal capacity of CD117^+^/CD133^+^ HNMM subpopulations was confirmed using a cell viability assay. An equal number of CD117^+^/CD133^+^ or CD117^−^/CD133^−^ HNMM cells derived from either P1, P2 or P3 were seeded for one week before the assessment of total cell growth. Data obtained from cell viability assays (Figure 1D) demonstrate that CD117^+^/CD133^+^ HNMM subpopulations have growth advantages over their matched CD117^−^/CD133^−^ HNMM subpopulations. To investigate the migration potency of CD117^+^/CD133^+^ HNMM subpopulations, both CD117^+^/CD133^+^ and CD117^−^/CD133^−^ HNMM subpopulations were allowed to grow for 24 h before the scratching was performed and then images of the scratched area were taken at 48 h (Figure 1E). An acceleration of wound healing was demonstrated 48 h after scratching of the cultured CD117^+^/CD133^+^ subpopulations, compared to the corresponding CD117^−^/CD133^−^ subpopulations. This is evidence of the migratory potential of the CD117^+^/CD133^+^ subpopulations.

### 3.2. Resistance of CD117^+^/CD133^+^ HNMM Cells to Imatinib Is Associated with the Activation of PI3K

**Figure 2:**
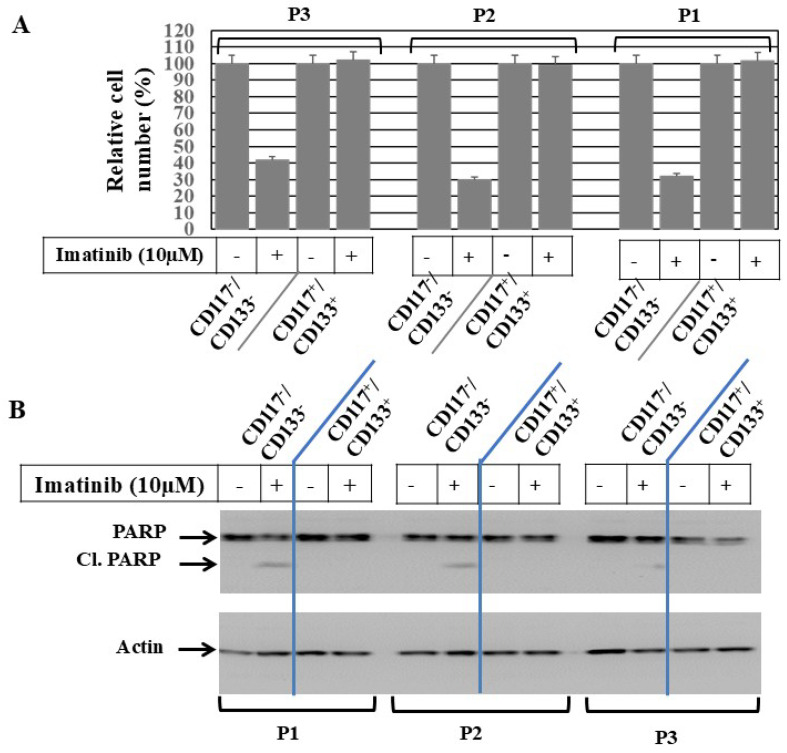
**CD117^+^/CD133^+^ HNMM cells conferring resistance to c-Kit inhibitor.** (**A**) Data of cell viability assay demonstrates the inhibition of cell growth of CD117^−^/CD133^−^, but not CD117^+^/CD133^+^ subpopulations, derived from HNMM specimens, following treatment with imatinib at indicated concentration for a period of 48 h. Results are expressed as mean ± SD from three separate experiments performed in triplicate. (**B**) Western blot analysis demonstrates imatinib-induced PARP cleavage in CD117^−^/CD133^−^, but not in CD117^+^/CD133^+^ cells derived from different specimens. Actin was used as internal control for loading and transfer. Data is representative of three independent experiments performed separately.

To investigate whether imatinib, the specific inhibitor of c-Kit, can influence the cell viabilty of CD117^+^/CD133^+^ subpopulations derived from HNMM patients with active c-Kit mutations, CD117^+^/CD133^+^ and their matched CD117^−^/CD133^−^ subpopulations were allowed to grow for 24 h under recommended conditions before treatment with imatinib (10 µM) for 48 h. Then, the viabilty of treated and control cells were assessed using MTT assay. The inhibition of cell viability (Figure 2A) was noted in CD117^−^/CD133^−^, but not in CD117^+^/CD133^+^ cells—an evidence for the resistance of CD117^+^/CD133^+^ cells to imatinib. To determine whether imatinib-induced inhibition of CD117^−^/CD133^−^ cells is mediated by an apoptotic mechanism, the total protein lysate of treated and control cells was subjected to the analysis of the apoptotic marker, PARP, by Western blot. The Western blot data (Figure 2B) demonstrates cleavage of PARP in CD117^−^/CD133^−^ cells, but not in CD117^+^/CD133^+^ cells, in response to treatment with imatinib. This provides evidence of CD117^+^/CD133^+^ cells’ resistance to imatinib.

**Figure 3:**
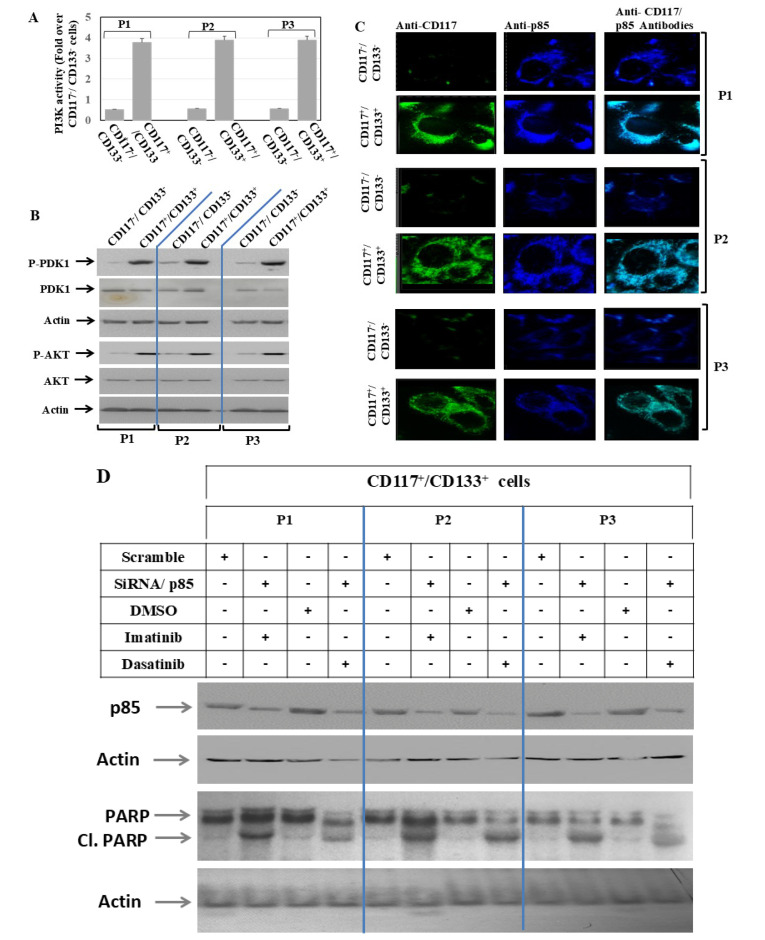

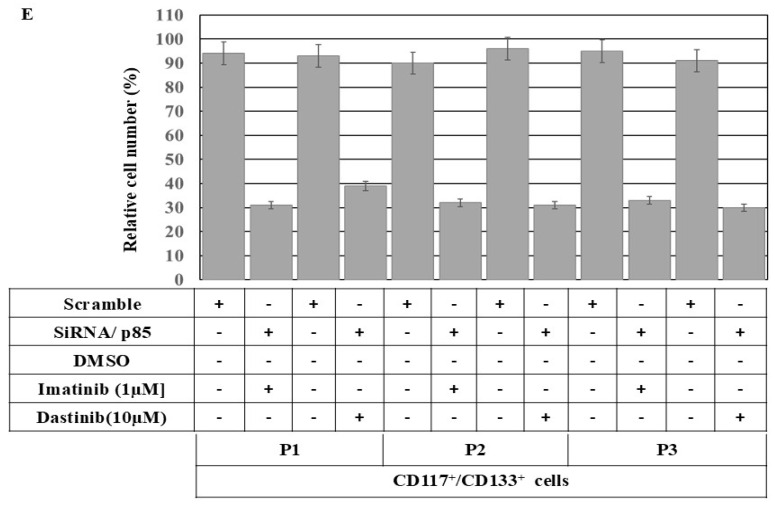
**Kinase assay.** (**A**) PI3K activity of CD117^+^/CD133^+^ versus CD117^−^/CD133^−^ cells derived from HNMM specimens P1, P2 and P3 using PI3K ELISA assay. Values are normalized to match CD117^+^/CD133^+^ cells. Results are expressed as mean ± SD from three separate experiments. (**B**) **Western blot analysis**: Data of Western blot analysis demonstrate the phosphorylation of both PDK1 and AKT proteins in CD117^+^/CD133^+^, when compared with those of their matched CD117^−^/CD133^−^ cells. Total PDK1 and AKT proteins and actin were used as internal control for loading and transfer. Data is representative of three independent experiments performed separately. (**C**) **Immune fluorescence staining**: Colocalization of c-Kit (green) and p85 (blue) in CD133^+^/CD117^+^ and CD133^−^/CD117^−^ cells derived from HNMM specimens. Colocalization of c-Kit and p85 is demonstrated (turquoise). Data are representative of three independent experiments. (**D**) **Western blot analysis**: Data of Western blot demonstrates the cleavage of PARP in CD117^+^/CD133^+^ cells derived from either P1, P2 or P3 specimens by the indicated inhibitors following the knockdown of p85 by its specific siRNA. Actin was used as an internal control for loading and transfer. Data are representative of three independent experiments. (**E**) **Cell viability assay**: Data of cell viability assay demonstrates the knockdown of p85 protein by its specific siRNA significantly inhibits the growth of CD117^+^/CD133^+^ cells in response to the treatment with the indicated inhibitors. Results are expressed as mean ± SD from three separate experiments performed in triplicate.

p85 is the crucial regulatory subunit of class IA phosphoinositide 3-kinase (PI3K), which acts as a molecular switch and balances PI3K/Akt signaling for cell growth, survival and metabolism [31]. P85 acts as a negative regulator by sequestrating p110 subunits and as a positive regulator by controlling PTEN phosphatase [19]. The N-terminal region of p85 binds and activates PTEN, thus effectively suppressing the activation of the PI3K signaling pathway, which is crucial for tumor suppression [42]. P85 acts as a negative regulator by sequestrating p110 subunits and as a positive regulator by controlling PTEN phosphatase [43]. To investigate whether the resistance of CD117^+^/CD133^+^ HNMM cells is regulated by the c-Kit signal to the PI3K pathway, the CD117^+^/CD133^+^ and their matched CD117^−^/CD133^−^ HNMM cells were allowed to grow for 48 h. Then, the total cell lysate was prepared for assessment of PI3K activity by ELISA. Data obtained from ELISA (Figure 3A) demonstrate the elevation of the basal PI3K activity in CD117^+^/CD133^+^ HNMM, when compared to those observed in CD117^−^/CD133^−^ cells. Next, we investigated whether the elevated activation of PI3K in the CD117^+^/CD133^+^ cells is associated with the activation of PDK1 and/or AKT; the CD117^+^/CD133^+^ and CD117^−^/CD133^−^ cells were allowed to grow for 48 h. Subsequently, the total cell lysates from control and treated cells were analyzed by Western blot for the expression and phosphorylation levels of both PDK1 and AKT. Western blot analysis (Figure 3B) reveals that the elevated activation of PI3K is corelated with the phosphorylation of PDK1 and AKT proteins in CD117^+^/CD133^+^, when compared to those observed in CD117^−^/CD133^−^ cells. This suggests an important role for PI3K in the modulation of cell growth and resistance of CD117^+^/CD133^+^ cells. The CD117^+^/CD133^+^ and CD117^−^/CD133^−^ cells were allowed to grow for 48 h under normal cell culture conditions. Then, the cells were probed for immune fluorescence (IF) staining using anti-CD117 or anti-p85 antibodies. Data of IF staining (Figure 3C) demonstrate the expression of CD117 (green) in CD117^+^/CD133^+^ cells, but not in CD117^−^/CD133^−^cells, while the detection of p85 (blue) was noted in both CD117^+^/CD133^+^ and CD117^−^/CD133^−^ cells. Merging CD117 (green) and p85 (blue) yielded a turquoise color, suggesting co-localization of p85 with CD117 proteins.

To investigate the effect of the suppression of the p85 protein by its specific siRNAs on the growth and resisance of CD117^+^/CD133^+^ cells, the cells were allowed to grow for 24 h before transfection with specific p85 siRNAs. Twenty-four hours post transfection, cells were treated with the recommended concentration of imatinib (10 µM) or dasatinib (1 µM) for 48 h. DMSO was used as a negative control. Treated and control cells were subjected to Western blot analysis and a cell viability assay. We anaylzed the expression of the p85 protein in control and siRNA transfected cells to assess the efficiency of siRNA-induced suppression of the p85 protein. Data of Western blot analysis (Figure 3A) demonstrate marked reduction in p85 expression in all transfected cells, when compared to control cells challenged with the control siRNA (Scramble). Although the treatment with DMSO did not demonstrate a significant effect on the cleavage of PARP, the suppression of p85 by its specific siRNA was found to overcome the resistance of CD117^+^/CD133^+^ cells to either imatinib or dasatinib inhibitors as evidenced by PARP cleavage (Figure 3D). This suggests that the destruction of the PI3K pathway is essential to sensitize CD117^+^/CD133^+^ cells to the specific inhibitors of c-Kit. Next, we confirmed the Western blot data by analysis of the cell viabilty of treated and control cells. As expected, data of cell viabilty assay (Figure 3E) demonstrate that the supression of p85 by its specific SiRNA is found to significantly reduce cell growth of CD117^+^/CD133^+^ cells in response to the treatment with either imatinib or dasatinib.

### 3.3. Suppression of p53 and Activation of NF-κB in CD117^+^/CD133^+^ HNMM Cells

**Figure 4:**
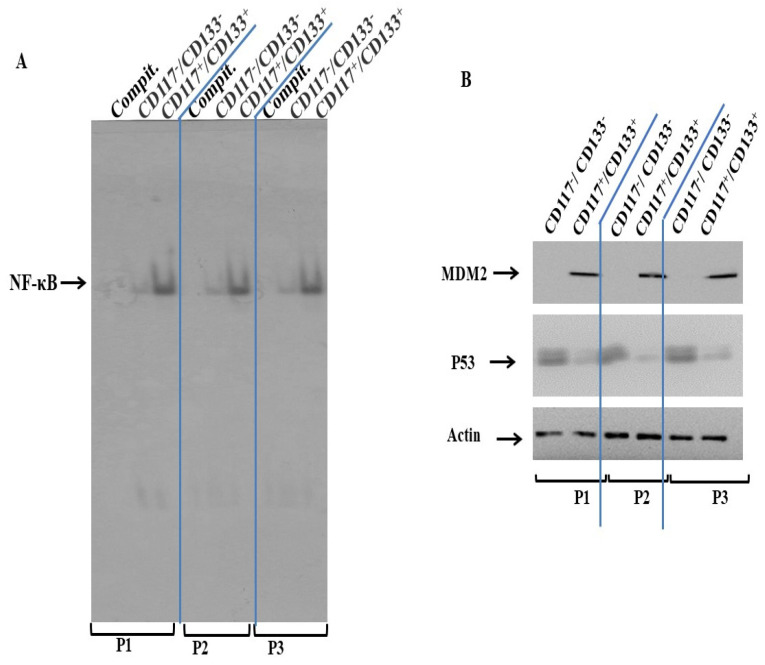
(**A**) **EMSA**: Data demonstrates the DNA-binding activity of the transcription factor NF-κB in CD117^+^/CD133^+^ and CD117^−^/CD133^−^ cells. Competitor (Compit.) was used as control for binding specificity. Data is representative of three independent experiments performed separately. (**B**) **Western blot**: Wesern blot analysis demonstrates the basal expression of both MDM2 and p53 proteins in CD117^+^/CD133^+^ and CD117^−^/CD133^−^ cells derived from either P1, P2 or P3 specimens. Actin was used as an internal control for loading and transfer. Data representative of three independent experiments performed separately.

The role of PI3K and its downstream signaling in the regulation of MDM2, p53 and the NF-κB-dependent pathway has been established [19]. We investigated whether the suppression of p53 and the activation of NF-κB are determined by the elevated activation of PI3K and its down stream pathways. CD117^+^/CD133^+^ and CD117^−^/CD133^−^ subpopulations derived from P1, P2 or P3 MM sampels were subjected for the extraction of total cell lysates and nuclear extracts to perform Western blot analysis and EMSA, respectively. The analysis of MDM2 protein, the negative regulator of p53 in both CD117^+^/CD133^+^ and CD117^−^/CD133^−^ cells (Figure 4A), reveals the suppression of the MDM2 protein in CD117^−^/CD133^−^ cells when compared to the basal level in CD117^+^/CD133^+^ cells, while the expression level of the p53 protein was markedly elevated in CD117^−^/CD133^−^, when compared to those in CD117^+^/CD133^+^ cells. Also, the analysis of the DNA-binding activty of the transcription factor NF-κB using the nuclear extracts of CD117^−^/CD133^−^ and CD117^+^/CD133^+^ cells by EMSA (Figure 4B) demonstrates the activation of NF-κB in CD117^+^/CD133^+^, but not in CD117^−^/CD133^−^ cells.

### 3.4. Regulation of CD117^+^/CD133^+^ HNMM Cells Growth and Resistance Are PI3K-Dependent Mechanisms

**Figure 5:**
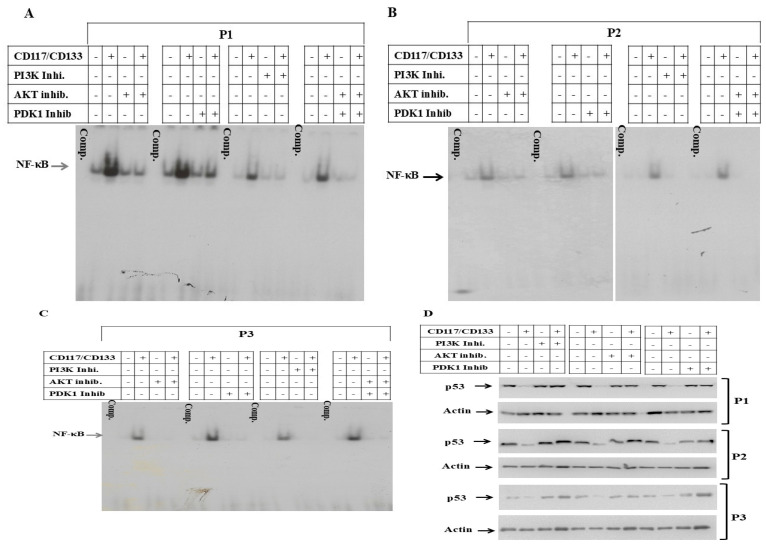

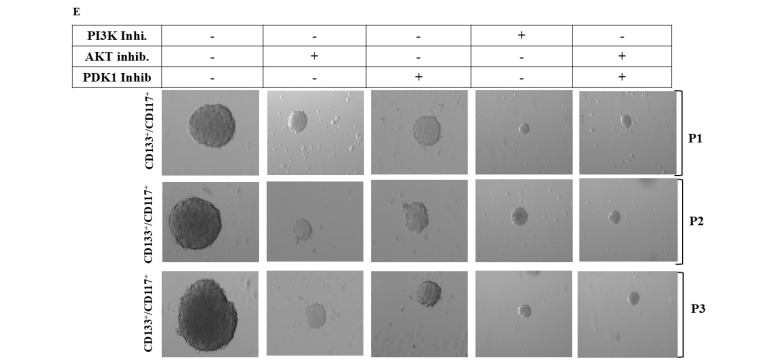
**EMSA**: Data of EMSA analysis demonstrate the inhibition of DNA-binding activity of NF-κB following the treatment of CD117^+^/CD133^+^ HNMM cells derived from HNMM speciemens of P1 (**A**), P2 (**B**) and P3 (**C**) with the inhibitors of PI3K (LY294002) at a concentration of 10 µM, AKT (AKT inhibitor VIII) at a concentration of 200 nM and PDK1 (BX517) at a concentration of 1 µM. Data are representative of three independent experiments performed separtately. (**D**) **Western blot**: Data of Western blot demonstrate the recovery of the suppressed p53 protein in response to the treatment of CD117^+^/CD133^+^ HNMM cells with the recommended concentration of PI3K, AKT or PDK1 inhibitors. Actin was used as an internal control for loading and transfer. Data are representative of three indpendent experiments performed seperatly. (**E**) **Single-cell sphere formation assay**: Data of spheroid formation assay demonstrates the inhibition of CD117^+^/CD133^+^ HNMM cells to form in vitro tumor-like spheres in response to the treatment with PI3K, AKT or PDK1 inhibitors. Images were captured on day 14. Data is representative of three independent experiments performed separately.

The activation of PI3K was assessed to determine if it is responsible for the sustained suppression of p53 proteins and elevated activation of NF-κB. This would support the hypothesis that the suppression of p53 mediates the maintenance of stem cell properties in CD117^+^/CD133^+^ cells, as well as their resistance to anticancer agents. The CD117^+^/CD133^+^ and their matched CD117^−^/CD133^−^ cells were allowed to grow for 24 h prior to the treatment with the inhibitors of PI3K, AKT and PDK1 for 48 h. Consequently, treated and control cells were subjected to the spheroid formation assay or the extraction of total protein and nuclear extracts for Western blot analysis and EMSA, respectively. The analysis of the binding activity of NF-κB by EMSA in both CD117^+^/CD133^+^ and CD117^−^/CD133^−^ cells (Figure 5A–C) demonstrates the reduction in the activity of NF-κB in CD117^+^/CD133^+^ and CD117^−^/CD133^−^ cells by the inhibitor of PI3K, AKT and PDK1. This suggests there is an essential role for PI3K, AKT and PDK1 in the regulation of NF-κB activation in CD117^+^/CD133^+^ cells. The analysis of the total cell lysates of treated and control CD117^−^/CD133^−^ and CD117^+^/CD133^+^ cells (Figure 5D) demonstrates the recovery of PI3K, AKT or PDK1 inhibitor-induced suppression of the p53 protein in CD117^+^/CD133^+^ cells, suggesting an essential role for PI3K and its downstream pathways PDK1 and AKT in the regulation of p53 expression in CD117^+^/CD133^+^ cells. It was then necessary to confirm whether the inhibition of the PI3K/AKT and PI3K/PDK1 axis can influence the self-renewal capacity of CD117^+^/CD133^+^ HNMM cells. CD117^+^/CD133^+^ cells were seeded at a concentration of a single cell, then the cells were allowed to grow in a serum-free, growth-factor-supplemented, anchorage-independent environment first for 48 h before the treatment with the inhibitors of PI3K (LY294002), AKT (AKT inhibitor VIII) and PDK1 (BX517). Data of the spheroid formation assay (Figure 5E) reveal that the treatment of CD117^+^/CD133^+^ cells with either PI3K, AKT or PDK1 inhibitors was found to negatively influnce the ability of CD117^+^/CD133^+^ cells to form spheres, when compared with control cells. These data confirm the role of PI3K, PDK1, and AKT in regulating the ability of CD117^+^/CD13^+^ cells to form spheres.

### 3.5. Disruption of CD117 Signal Transduction to PI3K Delays the Growth of CD117^+^/CD133^+^ Subpopulation in Mouse Xenografts

**Figure 6:**
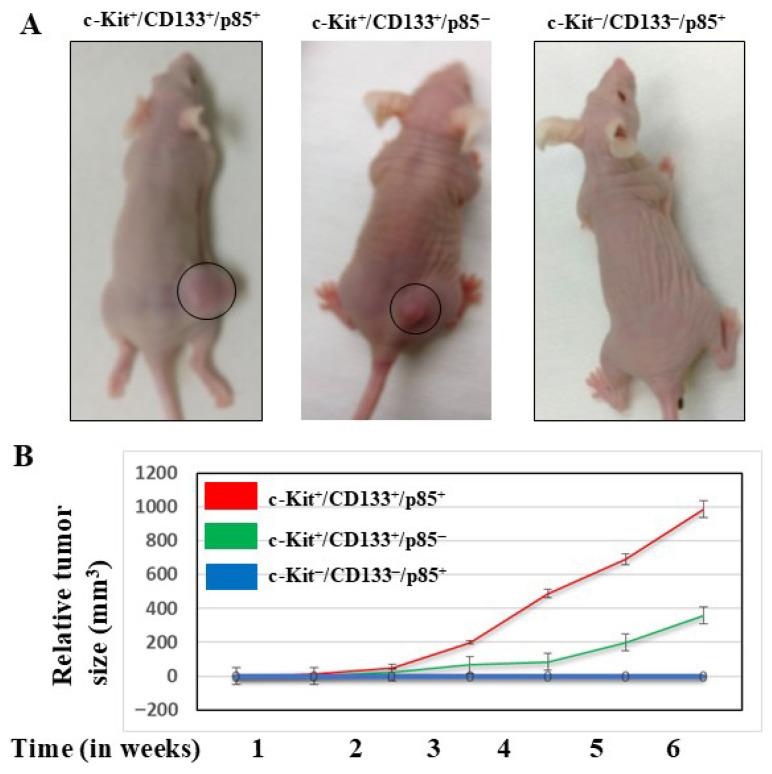
Tumor growth in mice bearing CD117^+^/CD133^+^/p85^+^, CD117^+^/CD133^+^/p85^−^ and c-Kit^−^/CD133^−^/p85^+^ HNMM subpopulations. (**A**) HNMM subpopulations (1 × 10^5^ cells) were implanted in mice groups, and the pictures were taken six weeks post implantation. (**B**) Tumor size was measured at regular time intervals up to 6 weeks.

We evaluated the role of the p85 protein as a mediator for the transmission of CD117 signal transduction to the PI3K pathway using a xenograft model for CD117^+^/CD133^+^ subpopulations. Groups of mice were Xeno-transplanted with CD117^+^/CD133^+^/p85^+^, CD117^+^/CD133^+^/p85^−^, CD117^−^/CD133^−^/p85^−^ HNMM^−^ melanoma subpopulations subcutaneously in the right flank. The tumor first appeared and continued to grow two weeks after inoculation in mice bearing CD117^+^/CD133^+^/p85^+^ cells, and three weeks after inoculation in groups of mice with CD117^+^/CD133^+^/p85^−^ cells; however, this was not the case in groups of mice with CD117^−^/CD133^−^/p85^−^ cells, even six weeks after inoculation (Figure 6A). The measurement of tumor growth rate over 6 weeks (Figure 6B) revealed the appearance of the tumor in mice bearing CD117^+^/CD133^+^/p85^+^ cells was first noted two weeks post inoculation and grows rapidly thereafter, while in mice bearing CD117^+^/CD133^+^/p85^−^ cells it was first noted three weeks post inoculation and grows slowly. In contrast to the mice carrying CD117^+^/CD133^+^/p85^+^ or CD117^+^/CD133^+^/p85^−^ cells, no tumor growth was observed in the group of mice with CD117^−^/CD133^−^/p85^+^ cells. This suggests an important role of the p85 protein in modulating the CD117 signal to PI3K and its dependent pathways, PDK1/AKT/NF-κB and PDK/AKT/MDM2, to trigger self-renewal capacity and drug resistance in CD117^+^/CD133^+^/p85^+^ cells.

### 3.6. Disruption of CD117/c-Kit Signal Transduction to PI3K Pathway Enhances the Therapeutic Efficacy of Imatinib in CD117^+^/CD133^+^ HNMM Xenografts

**Figure 7:**
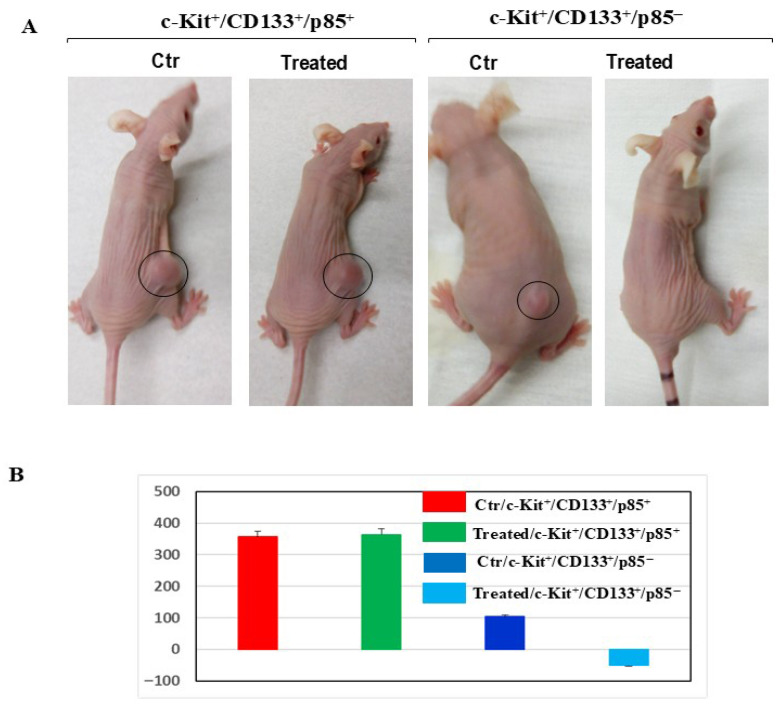
Mice were engrafted with CD117(c-Kit^+^)/CD133^+^/p85^+^, CD117(c-Kit^+^)/CD133^+^/p85^−^ HNMM subpopulations (1 × 10^5^ cells). (**A**) Mice groups were treated with imatinib by intraperitoneal injection at 50 mg/kg/mouse, twice a day, for 6 weeks and the picture was taken after the treatment has been completed. (**B**) Relative tumor volume of control and treated mice has been demonstrated. The measurement of the tumor volume in mice was performed by a digital caliper to determine length (l) and width (w) and the tumor volume was calculated using the formula: *V* = 1/2 (*l* × *w*^2^).

It was then important to determine if the knockdown of p85 in the CD117^+^/CD133^+^ subpopulation can overcome the resistance to imatinib in vivo. Two groups (five mice each) were Xeno-transplanted with CD117^+^/CD133^+^/p85^+^, and two groups (five mice each) were Xeno-transplanted with CD117^+^/CD133^+^/p85^−^ HNMM subpopulations subcutaneously in the right flank. The tumor growth was allowed to reach an elliptic area up to approximately 4–10 mm^2^. Treated mice received imatinib (50 mg/kg in 100 μL) and control mice received a vehicle (100 μL PBS) by intraperitoneal injection twice a day starting 48 h post inoculation. The tumor sizes were measured during the treatment period (Figure 7A). Four weeks after the treatment was finished, pictures of mice groups were taken (Figure 7B) to determine whether the knockdown and subsequently the disruption of CD117 signal transduction to PI3K can overcome HNMM resistance to imatinib. External measurements of the tumors (Figure 7A) indicate that the treatment with imatinib induces total tumor regression in mice bearing CD117^+^CD133^+^/p85^−^, but not in mice bearing CD117^+^/CD133^+^/p85^+^ cells, when compared to control groups. This data suggests that the knockdown of p85 and subsequently the disruption of the CD117^+^ signal to the PI3K pathway may offer a clinically relevant therapeutic strategy for the treatment of HNMM.

## 4. Discussion

In this present study, we demonstrate that tumor lesion of HNMM specimens, derived from mucosal melanoma of the hard palate bearing BRAF wild-typee, NRAS wild-type and Kit wild-type (P1), or bearing BRAF wild-type, NRAS wild-type and activating Kit mutation D820E (P2), or those derived from mucosal melanoma of nasal septum bearing BRAF wild-type, NRAS-activating mutation (Q61K) and KIT wild-type (P3) contain a small subset of cells with stemness properties. These CD117^+^/CD133^+^ subpopulations of either P1, P2 and P3 can self-renew and generate tumor-like spheres with progeny that possess the phenotypic heterogeneity of parental tumors in vitro and can form tumor in a xenograft model. The identified HNMM subpopulations are characterized by the expression of CD20, CD117, CD133 and 166 proteins, which are recognized as human stem cell markers [19,39]. Like with human CM [19,39,40], we demonstrate that HNMM cells bearing CD117^+^/CD133^+^ proteins isolated from surgery-resected HNMM tissues are able to generate tumor-like spheres during several successive generations in vitro and to form tumor in vivo, whereas their matched counterpart CD117^−^/CD133^−^ cells do not. In addition, we demonstrate that the stemness properties and drug resistance of the CD117^+^/CD133^+^ subpopulations are mediated by the CD117/c-Kit signal to PI3K and its downstream AKT/NF-κB, PDK1/AKT/NF-κB, AKT/MDM2, PDK1/AKT/MDM2 pathways. Thus, Kit-dependent activation of PI3K and its downstream pathways lead to sustained activation of NF-κB and expression of MDM2-dependent destabilization of the p53 protein. The sustained activation of NF-kB seems to contribute to the increased expression of the mast cell growth factor (stem cell factor (SCF)) that results in continuous activation of c-Kit and thereby triggers the activation of PI3K and its dependent pathways leading to tumor progression and treatment resistance.

The transcription of mast cell growth factor SCF (stem cell factor) is upregulated under inflammatory conditions and its transcriptional regulation is an NF-κB-dependent mechanism [44]. Therefore, the constitutive activation of NF-kB in the CD117^+^/CD133^+^ subpopulation is expected to trigger the upregulation of SCF, which in turn has the potential to enhance the activation of CD117 in CD117^+^/CD133^+^ which is mediated by a paracrine-dependent mechanism. Consequently, the activation of CD117 in the CD117+/CD133+ subpopulation by SCF leads to the phosphorylation of CD117 at the Tyr 721 residue. The phosphorylated Tyr-721 residue in the cytoplasmic domain of CD117 can thus recruit P85 to trigger the activation of PI3K and its downstream signaling pathways, thereby maintaining the proliferation of CD117^+^/CD133^+^ cells and their resistance to anticancer agents. Our data suggests that the downregulation of p85 sensitizes the CD117^+^/CD133^+^ subpopulations to treatment with imatinib both in vitro and in vivo.

Although the existence and the mechanistic role of CSCs in tumor progression and drug resistance have been widely discussed in a variety of tumor types including CM [16,19,40], less studies have been performed on the melanoma subtype MM [45,46].

Our data indicates that the CD117+/CD133+ subpopulation with BRAF wild-type and NRAS wild-type, which does not harbor D820E, a secondary mutation in Exon 17, as demonstrated in the tumor lesion (P1) or a CD117+/CD133+ subpopulation with BRAF wild-type, NRAS wild-type, and the activating KIT mutation (D820E), as detected in the tumor lesion (P2) or a CD117+/CD133+ subpopulation with wild-type BRAF as well as the activating NRAS mutation (Q61K), which, however, does not harbor the D820E mutation, as detected in the tumor lesion (P3) confers resistance to Kit inhibitors including imatinib. This observation suggests that the resistance of HNMM does not only result in activating the KIT D820E mutation localized to Exon 17 but can also be attributed to other activating mutations localized to Exons 11 and 13. The destruction of the PI3K pathway was found to increase the sensitivity of the CD117^+^/CD133^+^ subpopulation derived from the different mucosal melanoma lesions. Thus, it is expected that CD117-mediated activation of the PI3K pathway is not the consequences of mutations located to Exon 17 such as D820E but may also result from other mutations located to Exons 11 and 13. However, the detection and functional analysis mutations, whose occurrence is common in Exons 11 and 13, may clear the clinical relevance of our finding. Thus, based on the data reported by Kaveti et al. [44], our data indicates that the resistance of the CD117^+^/CD133^+^ subpopulations is not determined only by the KIT mutation D820E on Exon 17, but possibly also by other mutations in Exons 11 and 13.

In this study, we identify and functionally analyze HNMM subpopulations, derived from patients’ specimens displaying stemness properties both in vitro and in vivo. In addition, we provide insight into the mechanisms by which CD117^+^/CD133^+^ cells can maintain their stemness properties and evade drug toxicity. Similar to our previous studies on CSCs of human CM [19,39,40], we analyzed the stem cell properties of HHMM subpopulations in vitro as well as under in vivo-like conditions. We demonstrate that the progression and treatment resistance of HNMM is mediated by the CD117 signal to PI3K and its dependent pathways. We also confirm that the identified HAMM subpopulations exhibit the same biological behavior as CSCs from a variety of other tumor types. In addition to the expression of the stem cell markers (e.g., CD20, CD117, CD133 and CD166), our findings show that HNMM subpopulations are characterized by their self-renewal capacity, the ability to form tumor-like spheres in vitro and to develop tumors in vivo, and in contrast to the mice carrying CD117^+^/CD133^+^/p85^+^ or CD117^+^/CD133^+^/p85^−^ cells, no tumor growth was observed in the group of mice with CD117^−^/CD133^−^/p85^+^ cells. This suggests an important role of the p85 protein in modulating the CD117 signal to PI3K and its dependent pathways (PDK1/AKT/NF-κB and PDK/AKT/MDM2) to maintain self-renewal capacity and to confer resistance to c-Kit inhibitors.

The increased frequency of Kit aberrations in MMs has been reported [25,47]. Although c-Kit does not contribute to the etiopathogenesis of most CMs, Kit aberrations in MM are of pathogenic importance [25].

Imatinib, the inhibitor of c-Kit, belongs to a class of tyrosine kinase inhibitors/c-Kit blockers that have been approved for treatment of many cancers with Kit mutations [48,49]. Our results show that the resistance of CD117^+^/CD133^+^ cells to Kit inhibitors is PI3K-dependent activation and the disruption of the CD117/c-Kit signal to PI3K through the knockdown of p85 is essential to block the formation of tumor-like spheres in vitro and to delay tumor growth in vivo. Also, we found that the destruction of the Kit signal to PI3K is essential to overcome the resistance of CD117^+^/CD133^+^ cells to imatinib, both in vitro and in vivo.

The PI3K/PDK1/AKT pathway represents an aberrant pathway responsible for the promotion of cell survival and inhibition of apoptosis [50,51]. Growing evidence indicates that the activation of the PI3K signaling pathway is mediated by either direct or indirect mechanisms. Direct activation occurs via the interaction of p85 with c-Kit at a tyrosine 721 (Tyr^721^) residue [52,53], while indirect activation is mediated through scaffold protein Gab2 and adapter protein Grb2 [54,55]. The phosphorylation of c-Kit by its ligand, SCF, or at the Tyr^721^ residue is essential to recruit p85 to enhance PI3K activation that in turn mediates the activation of downstream signaling pathways such as PDK1 and AKT to trigger tumor growth and evade drug toxicity [56,57].

The observed levels of kinase activity of the PI3K pathway in CD117^+^/CD133^+^ cells, but not in their matched CD117^−^/CD133^−^ cells indicates that the activation of PI3K and its dependent pathways in HNMM cells is mediated via the interaction between CD117 and p85 proteins. In addition to slowing down tumor-like sphere formation in vitro and delaying tumor growth in vivo, we found that the knockdown of p85 in CD117^+^/CD133^+^ cells sensitizes CD117^+^/CD133^+^ cells to imatinib both in vitro and in vivo. Like CM [19,58], our results in HNMM underscore the crucial role of the PI3K signaling pathway not only in the development and progression of the disease, but also in controlling drug toxicity. The frequent indications of data similarities between HAMM and CM suggest the general applicability of these findings, regardless of the tissue origin of the melanoma, whether cutaneous or mucosal. According to this, the destabilization of p53 plays a crucial role in the development of drug resistance in many types of tumors, including CM and MM [19,59].

As is well known, p53, as a transcription factor, plays a crucial role in determining cell fate through various mechanisms, including cell growth arrest [60,61], cellular senescence, and DNA repair or apoptotic signal pathways [60,61].

The role of PDK1 in regulating melanoma progression and drug resistance is also well-documented; similar mechanisms have been described for other types of cancer [62,63]. PDK1 is not only involved in regulating signaling pathways such as PI3K/PDK1/AKT but also plays an important role in tumor progression and drug resistance [64,65,66].

AKT is serine/threonine kinase that is involved in several cellular functions including glucose metabolism, cell survival, growth, and proliferation [19,67]. In many types of cancer, including melanoma, the PI3K/PDK1/AKT signaling pathway plays an important role in tumor progression through the activation of the transcription factor nuclear factor-κB (NF-κB) [64,68], and in increasing drug resistance through the phosphorylation of MDM2, which leads to the destabilization of p53 and the activation of NF-κB [69,70].

Preferentially expressed antigen in melanoma (PRAME) is a cancer testicular antigen (CATs) that is frequently expressed in melanoma cells and serves as a valuable diagnostic marker to differentiate malignant from benign mucosal melanocytic tumors [1,2]. While PRAME shows a strong, diffuse nuclear staining pattern in malignant tumors, it is rarely or only focally present in benign nevi [3,4]. Therefore, PRAME not only serves as a potential prognostic marker in mucosal melanoma, as its high expression usually correlates with a more aggressive tumor phenotype and a worse prognosis, but is also considered a potential target for immunotherapy [5,6,7]. In addition to its expression in various malignancies, PRAME expression is associated with tumor aggressiveness and self-renewal in cancer stem cells [8,9]. PRAME in mucosal melanomas has been reported to be high and is usually associated with a poor prognosis and a more aggressive disease course [10,11]. Besides the expression of PRAME in various malignancies, but not in normal somatic tissues, PRAME has been established to play key roles in pluripotent and germ cell differentiation. It acts as a stem cell marker and transcription regulator that is acting downstream of SOX17 [71,72]. Although a subset of PRAME-negative malignant tumors was identified, especially located in the palatal area of the hard and soft palate [73], the high PRAME expression was associated with melanocytic tumors including mucosal melanoma of nasal cavity/nasal septum/turbinates nasopharynx, and the maxillary sinus [73,74]. Furthermore, it has been reported that the PRAME-induced promotion of tumor progression, immune escape, and the metastasis of squamous carcinoma cells is mediated by a mechanism dependent on the PI3K/AKT/mTOR signaling pathway [75]. Therefore, it is assumed that PRAME expressions may contribute to the activation of the PI3K signaling pathway in HNMM, thereby driving tumor progression and drug resistance. In the present study, we investigated the significant role of the PI3K signaling pathway in regulating the maintenance and drug resistance of the HNMM subpopulation CD117^+^/CD133^+^.

**Figure 8:**
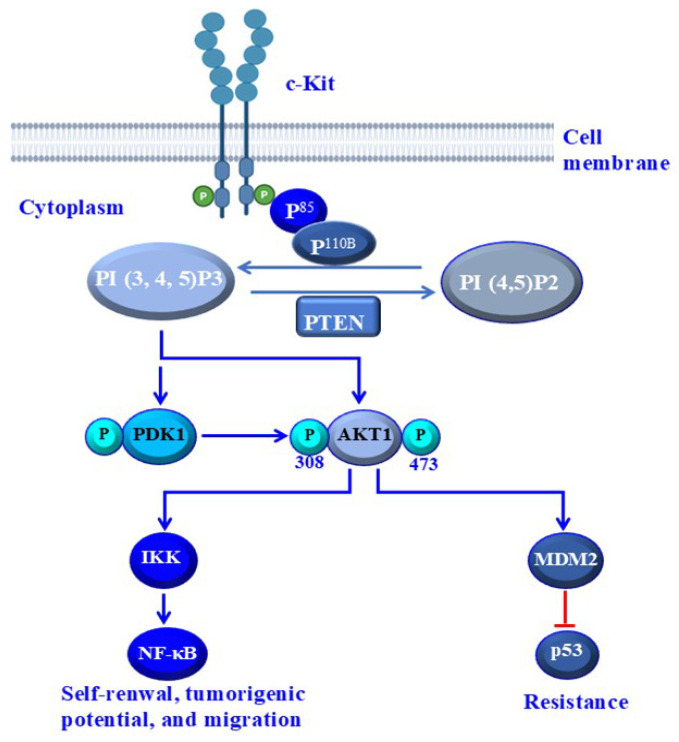
The proposed model outlines the possible mechanisms, which are essential for the maintenance of stemness properties and drug resistance of HNMM cells. The activation of PI3K and its downstream signaling pathways, AKT/NF-κB, PDK1/AKT/NF-κB, AKT/MDM2, PDK1/AKT/MDM2, are mediated by direct or indirect activation-dependent mechanisms. Direct mechanisms occur via the interaction of p85 with c-Kit at a Tyr^721^ residue to initiate the activation of multiple pathways including PI3K/PDK1/AKT and PI3K/AKT pathways to trigger the activation of NF-κB to maintain stemness properties and self-renewal capacity. The activation of PI3K/PDK1/AKT/MDM2 and PI3K/AKT/MDM2 pathways is essential to destabilize the p53 protein and subsequently enable HNMM cells to evade drug toxicity.

In summary, we have shown that HNMM, like CM, contains genetically divergent subpopulations of CSCs in a dormant state, characterized by the expression of stem cell markers, such as CD20, CD117, CD133 and CD166 proteins. Thus, CD117 signals to PI3K/PDK1/AKT/NF-κB and PI3K/PDK1/AKT/MDM2 pathways are essential to preserve stem cell properties and neutralize drug toxicity in HHMM as outlined in Figure 8. Collectively, these results add to the understanding of the biology of MM and may help identify potential therapeutic target(s) for the treatment of this rare tumor.

## Data Availability

The original contributions presented in this study are included in the article. Further inquiries can be directed to the corresponding author.

## Abbreviations

The following abbreviations are used in this manuscript:

HNMM	Head and neck mucosal melanoma
MM	Mucosal melanoma
CM	Cutaneous melanoma
CSCs	Cancer stem-like cells
